# A New Weighted Degree Centrality Measure: The Application in an Animal Disease Epidemic

**DOI:** 10.1371/journal.pone.0165781

**Published:** 2016-11-01

**Authors:** Luca Candeloro, Lara Savini, Annamaria Conte

**Affiliations:** Statistic and GIS department, Istituto Zooprofilattico Sperimentale dell’Abruzzo e del Molise ‘‘G. Caporale”, Teramo, Italy; Universite de Namur, BELGIUM

## Abstract

In recent years researchers have investigated a growing number of weighted heterogeneous networks, where connections are not merely binary entities, but are proportional to the intensity or capacity of the connections among the various elements. Different degree centrality measures have been proposed for this kind of networks. In this work we propose weighted degree and strength centrality measures (WDC and WSC). Using a reducing factor we correct classical centrality measures (CD) to account for tie weights distribution. The bigger the departure from equal weights distribution, the greater the reduction. These measures are applied to a real network of Italian livestock movements as an example. A simulation model has been developed to predict disease spread into Italian regions according to animal movements and animal population density. Model’s results, expressed as infected regions and number of times a region gets infected, were related to weighted and classical degree centrality measures. WDC and WSC were shown to be more efficient in predicting node’s risk and vulnerability. The proposed measures and their application in an animal network could be used to support surveillance and infection control strategy plans.

## Introduction

Network analysis has been used as an explanatory tool to describe the evolution and spread of ideas and innovations in societies [1]; observed social dynamics can often be understood through the analysis of the social networks that underlie them. A network is a set of nodes (actors), that could be individuals, organizations, holdings, administrative units, connected by a set of ties, that can refer to friendship or communications or animal movements or trade.

Attention has been given to the nature of connections, particularly to properties such as symmetry and transitivity (whether the friend of a friend is a friend), which together provide measures of social cohesion [2,3]. In addition, measures of the importance of individuals have also been derived, from the simplest (such as the number of connections) to the highly complex (number of paths between other actors in which an individual features) [3,4].

Ties between nodes can be considered in different ways when analyzing network structures. A great number of studies have been conducted to find measures about the strength of connections and a variety of centrality measures (CM) have been developed for the quantification of the interconnectedness of actors [5–11]. Degree centrality (DC) represents the simplest CM and determines the number of direct contacts as an indicator of the a network node's interconnectedness. The advantage of DC is the relatively easy interpretability and communicability of the results [12]. It can be easily calculated because only what happens around a focal node is needed. Other measures of centrality have been proposed to consider global network structure, namely betweeness and closeness [6,13].

Most of the previous measures only concern networks based on the presence or absence of a tie between two nodes (binary network), and when applied on weighted networks (in which an attribute is used to weight the tie between nodes), a loss of information occurs [3,14].

An increasing number of studies have been focused on finding appropriate measures for weighted networks [5,15,16].

In weighted networks the degree centrality is calculated as the sum of weights assigned to the node’s direct connections and represents the node strength (Strength Centrality—SC). It is then based on tie weights and not on the number of ties. The disadvantage is that two nodes with the same strength, can be linked to a different number of nodes, and the initial information caught by DC is lost when SC is calculated. To overcome this disadvantage a tuning parameter has been defined to give relevance either to tie weights or number of ties alternatively [10].

In this work a new weighted DC (WDC) has been developed to account for tie weights distribution. This new measure has been applied to an animal movement network in Italy, where nodes are administrative units (NUTS 2 level) and ties are animal movements. Considering the same amount of links and the same amount of animals moved, in such a network it is possible to find different situations: an ‘equal’ number of animals is dispatched to linked nodes; a high number of animals is dispatched to a single linked node and only a few animals are distributed among others. While the DC is the same in these two cases, the developed WDC is lower in the last case providing a more proper measure of connectedness.

A number of studies have been carried out to analyse the networks of animal movements in order to predict the spread of diseases or to optimise the control and surveillance strategies in an outbreak situation [17–22]. These works showed the degree centrality measure (among the known classical network centrality measures) to have a better ability to detect the central role of holdings in the network of animal movements.

In this paper a disease spread has been simulated through the animal movement network, testing how WDC (both in terms of in-degree and out-degree) led to a better estimate of nodes’ vulnerability and infecting capacity (risk) than DC values.

## Material and Methods

**Fig 1:**
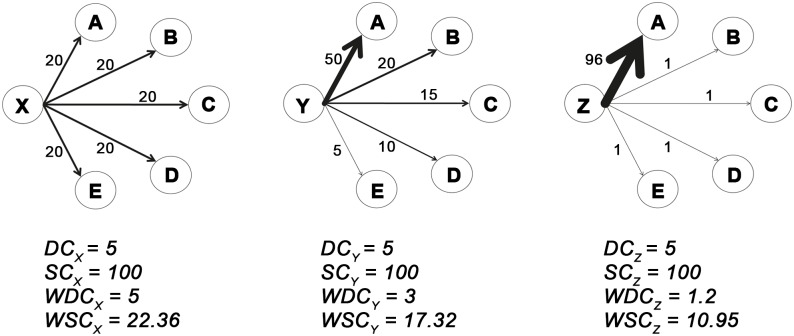
Three examples of networks where classical measures of degree and strength give same results. Three different situations with the same DC and SC are illustrated: three focal nodes (X, Y, Z) are connected to the same number of nodes (DC = 5) with the same nodes’ strength (SC = 100). The strength of each focal node is distributed among linked nodes in different ways: uniformly (X node) or to a fewer number of nodes (case Y and Z nodes).

To illustrate the concept underlying the new WDC calculation, three different situations with the same DC and SC are illustrated: three focal nodes (X, Y, Z) may be connected to the same number of nodes (DC = 5) with the same nodes’ strength (SC = 100), see Fig 1. But the strength of each focal node can be distributed among linked nodes in different ways: uniformly (in case of X node) or privileging only a fewer number of nodes (in case of Y and Z nodes). It is intuitive the need to reduce the degree of Y and Z nodes in relation to the fact that 85% and 96% of the weights concerns a relationship with only three and one of the linked nodes respectively.

The idea is to calculate a reducing factor (R) that doesn’t change the DC value when weights (w) are uniformly distributed (node X) and reduces the original DC value accordingly to the shift from the uniform condition (nodes Y and Z).

**Fig 2:**
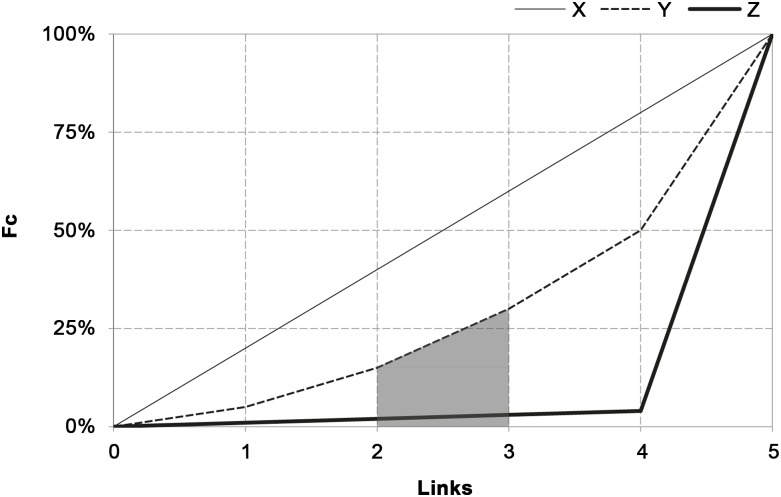
Empirical cumulative weights’ distribution (Fc) for nodes X, Y, Z. Graphical representation of the Area Under the Curve AUC_Fc_ of weights relative to nodes X, Y and Z of Fig 1. The AUC_Fc(3),_ the area related to the first 3 links of Y node is highlighted in grey.

The calculation method of the reducing factor (R) originates from the cumulative distribution of the percentage weights (Fc), for X, Y and Z nodes as illustrated in Fig 2.

The area under the Fc (AUC) decreases accordingly with the deviation from equal weight distribution. Under uniformly distributed weights (node X) F_c_ is a straight line, with constant slope equal to 1/DC and with AUC reaching the maximum. As F_c_ deviates from this straight line, the AUC drops down consequently.

The behavior of F_c_ allows to calculate R as the ratio between the AUC_Fc_ and AUC_max_:

$$R=\frac{AUC_{Fc}}{AUC_{max}}$$

WDC=R⋅DC

In such a way, when weights distribution is uniform AUC_Fc_ is equal to AUC_max_, R becomes 1 and WDC is equal to DC.

In this way the original number of links is reduced and a more reliable number of links is associated to each focal node.

As both SC and WDC can vary independently, the WSC is defined as their geometric mean, so that if SC (or WDC) remains constant and WDC (or SC) rises, then WSC assumes a higher value and vice versa:

$$WSC=\sqrt{SC\cdot WDC}$$

### Formulas in AUCmax and AUCFc calculation

AUC_max_ is the area under the straight line with equation $Fc=\frac{x}{DC}$, with x ranging from 0 to DC, thus:

$$AUC_{max}=\int_{0}^{DC}\frac{x}{DC}dx\;=DC/2$$

The AUC_Fc_ is the sum of the areas AUC_Fc(i)_ under segments with different slopes for each link *i*:

$$AUC_{Fc}=\sum_{i=1}^{DC}AUC_{Fc\left(i\right)}$$

To calculate the area AUC Fc(i) the equation of each segment (line) is needed: A straight line passing for 2 known points {(i-1); Fc(i-1)} and {i;Fc(i)}, is:

$$\frac{Fc\left(x\right)-Fc\left(i-1\right)}{Fc\left(i\right)-Fc\left(i-1\right)}=\frac{x-\left(i-1\right)}{i-\left(i-1\right)}$$

Than solving for Fc_(i)_:

$$Fc\left(x\right)-Fc\left(i-1\right)=\frac{\left(x-i+1\right)}{1}\cdot \left(Fc\left(i\right)-Fc\left(i-1\right)\right)$$

Considering that:

$$Fc\left(i\right)=\sum_{J=1}^{i}f_{j}$$

where:

$$f_{j}=\frac{w_{j}}{\sum_{i=1}^{DC}w_{i}}$$

The previous equation becomes:

$$Fc\left(i\right)-\sum_{J=1}^{i-1}f_{j}=\left(x-i+1\right)\cdot \left(\sum_{J=1}^{i}f_{j}-\sum_{J=1}^{i-1}f_{j}\right)$$

$$Fc\left(x\right)=\left(x-i+1\right)\cdot f_{i}+\sum_{J=1}^{i-1}f_{j}$$

Thus

$$AUC_{Fc\left(i\right)}=\int_{i-1}^{i}{\left(\left(x-i+1\right)\cdot f_{i}+\sum_{J=1}^{i-1}f_{j}\right)}_{dx}=\;\;\frac{f_{i}}{2}+\sum_{J=1}^{i-1}f_{j}$$

And

$$AUC_{Fc}=\sum_{i=1}^{DC}AUC_{Fc\left(i\right)}=\sum_{i=1}^{DC}\left(\frac{f_{i}}{2}+\sum_{J=1}^{i-1}f_{j}\right)=\frac{1}{2}\sum_{i=1}^{DC}f_{i}+\sum_{i=1}^{DC}\sum_{J=1}^{i-1}f_{j}=\frac{1}{2}+\sum_{i=1}^{DC}\sum_{J=1}^{i-1}f_{j}$$

As $\sum_{i=1}^{DC}f_{i}=1$

And being

$$\sum_{i=1}^{DC}\sum_{J=1}^{i-1}f_{j}=\sum_{i=1}^{DC-1}\sum_{J=1}^{i}f_{j}$$

$$AUC_{Fc}=\frac{1}{2}+\sum_{i=1}^{DC-1}Fc\left(i\right)$$

To compute the new weighted degree measure, a custom function in R [23] has been developed.

### Dataset and network representation

Data on cattle trade movement used in the present study are obtained from the Italian National Bovine Database (NBD). The database contains detailed data about movement of each animal. Each movement record reported the unique identification code of the animal, the code of the origin and destination holdings, and the date of movement. In addition the following attribute for each holding were exported from the database: the holding type (farm, slaughterhouse, market etc.), number of animals at the beginning of the year and the address. The dataset refers to the year 2009.

The network was built from the data movement by aggregating at region level. Movements towards slaughterhouses and to and from foreign countries have been removed from the dataset.

**Fig 3:**
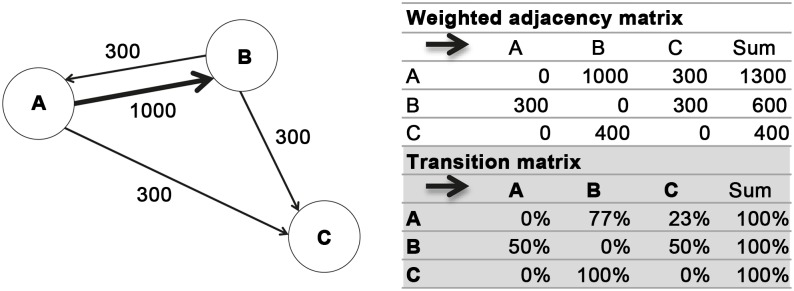
An example of weighted network and the related weighted adjacency matrix and transition matrix. The transition matrix shows how the number of animals that come out from a node is distributed to the nodes with which it is connected.

The resulting directed weighted network (IT2009) consists of 21 nodes (Italian regions) and 369 ties, where tie weight is defined by the number of live cattle moved from/to a region during the year. The network has been represented by an adjacency matrix A whose elements [A]_j,k_ indicate whether pairs of nodes are adjacent or not in the network. In the special case of a finite weighted network, the adjacency matrix stores directly edge weights [24]. A is a 21x21 matrix. An example of weighted network and the related weighted adjacency matrix is shown in Fig 3. In Table 1 the symbology adopted throughout the text to define a generic matrix A is reported.

**Table 1:** Symbology adopted throughout the text to define a generic matrix A.

Symbol	Definition
A	Matrix A
[A]_i,j_	Element in row i and column j of matrix A
A_i_	Row i of matrix A
A^j^	Column j of matrix A

### Model description

The disease spread on the network is modelled using a simple Susceptible-Infectious (SI) compartmental model [17,21,25]. Regions are the units of the process and are labelled as Susceptible or Infectious according to the stage of the disease. All regions are considered susceptible at the beginning of the simulations, except for the single seeding node whose prevalence of animals moved (percentage of infected animals moved) is set to 5%.

The considered model is a stochastic process where an infectious node can transmit the disease along its outgoing ties to its neighboring susceptible nodes that in turn become infected.

The model uses a daily framework and movements are supposed to be uniform through the year, hence daily number of animals moved are approximatively constant.

Defined the 21x2 matrix M, whose element [M]_j,c_ represents the number of outgoing animals from region j, with sanitary state *c* (where *c = 1* for infected an *c = 2* for susceptible animals), infected moved animals [M]_j,1_ from node *j* is supposed to be binomial distributed at each time-step t:

$${\left[M\right]}_{j,1}\sim Binomial\left(m_{j};\frac{i_{j}}{N_{j}}\right)$$

And

$${\left[M\right]}_{j,2}=m_{j}-{\left[M\right]}_{j,1}$$

Where:

*m*_*j*_ is the number of moved animals from node j

*i*_*j*_ is the number of infected animals in node j

*N*_*j*_ = *s*_*j*_ + *i*_*j*_ is the number of animals of node j derived from the total of susceptible (s_j_) and infected (i_j_) animals in node j. N_j_ is the population at 01/01/2009 for the first time step.

The number of infected animals moved from a node *j* towards its neighbors is calculated by a multinomial distribution of the number of infected animals moved from *j* towards a destination node and the percentage reported into the transition matrix T_:_

$$I_{j}\sim Multinomial\left({\left[M\right]}_{j,1};T_{j}\right)$$

Where I is the matrix whose elements [**I**]_j,k_ represents infected animals moved from region j to region k, and T is the row-standardized adjacency matrix ([**T**]_j,k_ = [**A**]_j,k_ /sum(**A**_j_)). **T** reflects how the number of animals outgoing from a node *j* is distributed to other nodes. An example of transition matrix is shows in Fig 3.

Similarly for susceptible animals moved:

$$S_{j}\sim Multinomial\left({\left[M\right]}_{j,2};T_{j}\right)$$

At each time step, population dynamic of node j is described by the following ODE’s system:

$$\{\begin{array}{c} \frac{ds_{j}}{dt}=sum\left(S^{j}\right)-{\left[M\right]}_{j,2}\\ \frac{di_{j}}{dt}=sum\left(I^{j}\right)-{\left[M\right]}_{j,1} \end{array}$$

Each simulation was iterate 100 times and repeated for 21 scenarios, using a different region as seeding node per each scenario and 365 time steps.

### Measures evaluation in terms of vulnerability and risk

The classical and the new weighted centrality measures have been calculated on the IT2009 network. Considering that the network is direct, both in and out degree measures have been calculated.

The spread model has been applied to the network to simulate infection across the regions. At each simulation the number of infected nodes (seeding site excluded) and the number of infected animals per region have been registered.

The model was run assuming two different population and movement conditions to better evaluate differences between classical and new measures in terms of risk and vulnerability:

1. in the first condition we assume that all nodes have the same population size (using a fixed value for each node set to 100000) and move the same number of animals (equal to 3,65% of population) and model results are used for WDC and DC evaluation (when nodes have the same strength the difference between WDC and DC is more evident and the ability of the new measure to capture the role of the node based only on the network structure is evaluated).
2. in the second condition the model employs real values of population and number of moved animals and model results are used for WSC and SC evaluation. Similarly a randomized network, obtained by randomizing (network) structure and by conserving the node's weight, was considered. Correlation between centrality measures and epidemic model results was evaluated through non-parametric Kendall’s Tau correlation coefficient.

## Results and Discussion

The classical and the new weighted centrality measures calculated on the IT2009 network dataset revealed different patterns when compared. The new weighted measures showed a significant difference in terms of nodes’ roles, not highlighted by the classical measures. In the case of a high network density, as IT2009, these differences are particularly evident (density values of region and holding level networks are 0.88 and 0.0000207 respectively),.

**Fig 4:**
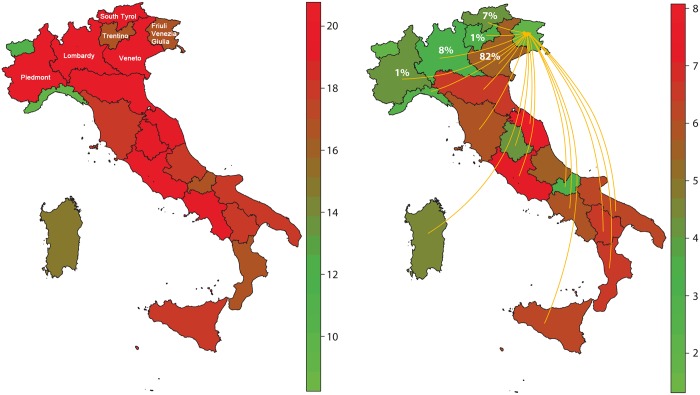
DC_IN_ and WDC_IN_ values for IT2009 data. The WDC_IN_ (on the right) shows a more evident variability among regions than DC_IN_ (on the left). The scale bars report the DC_IN_ and WDC_IN_ values for each region. In case of Friuli-Venezia-Giulia region, in the north-east part of Italy, the two measures are particularly different (DC_IN_ = 17 and WDC_IN_ = 1.73). Arrows representing in-going links are reported (17 regions), but only 5 regions already cover the 99% of the weights (f_*i*_ values are reported on the corresponding region).

Fig 4 shows the spatial distribution of DC_IN_ and WDC_IN_ values. The WDC_IN_ shows a greater variability between regions than DC_IN_ (the variation coefficient of WDC_IN_ is 0.36 and DC_IN_ is 0.16). Moreover the differences between northern and southern regions arise.

For example the Friuli-Venezia-Giulia region is one of the regions in which DC_IN_ and WDC_IN_ are particularly different (DC_IN_ = 17 and WDC_IN_ = 1.73). The WDC_IN_ value reflects that only few links cumulate up to 99% of the weights (just one link, Veneto region, has the 82% of the total weights).

**Fig 5:**
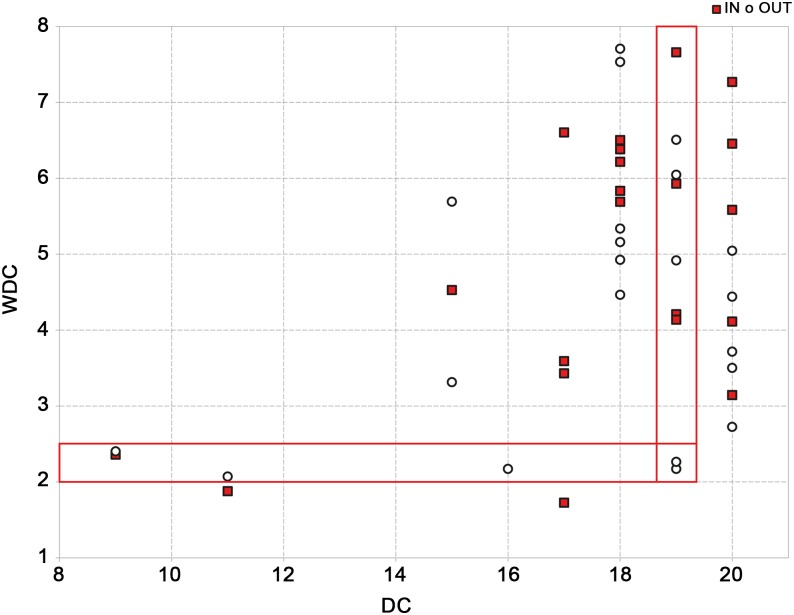
Correlation between the two measures DC and WDC of the IT2009 network. The correlation shows a moderate agreement between DC and WDC (0.55 ‘in’ and 0.31 ‘out’). For example, in 5 regions the same value of 19 for out-degree, is associated to a weighted out degree range of 2–7 and vice-versa 5 regions with values of weighted out-degree between 2 and 2.5, have values of out-degree ranging from 9 to 19.

As expected the correlation of the two measures is low as showed in Fig 5; for example, 5 regions that have the same value of 19 for out-degree, present a wide range of weight degree values between 2 and 7.

**Fig 6:**
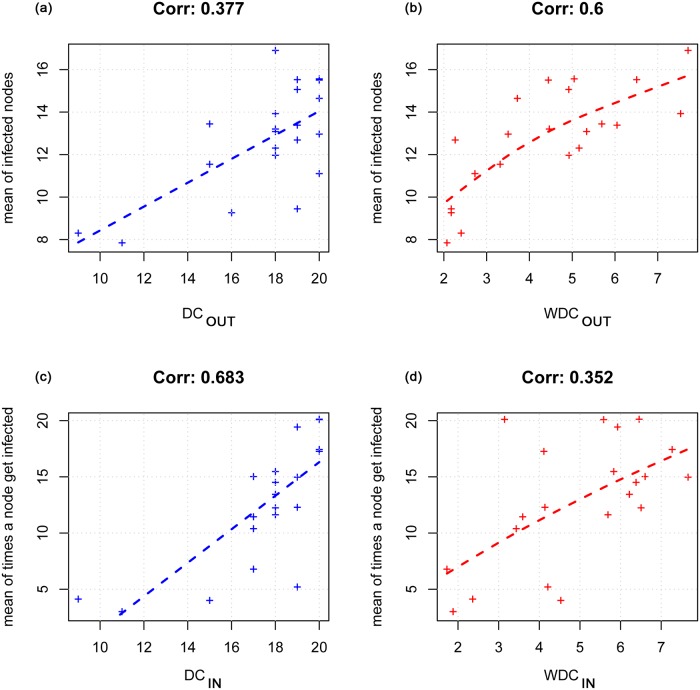
Correlation between simulation model results and degree centrality measures in the assumption that all nodes have the same population size. In the upper side of the figure the correlation between the node’s risk (the mean number of infected nodes) and DC_OUT_ (a) and WDC_OUT_ (b) is showed; the last two graphs report the correlation between node’s vulnerability (the mean number of times a node gets infected) and the DC_IN_ (c) and WDC_IN_ (d).

Fig 6 shows the correlation values between model results and degree centrality measures when it is assumed that all nodes have the same population size (first condition).

In the Fig 6(a) and 6(b) the correlation between the average number of infected nodes (node’s risk) and DC_OUT_ and WDC_OUT_ is showed; the last two graphs (c) and (d) report the correlation between the average number of times a node becomes infected (node’s vulnerability) and the DC_IN_ and WDC_IN_.

**Fig 7:**
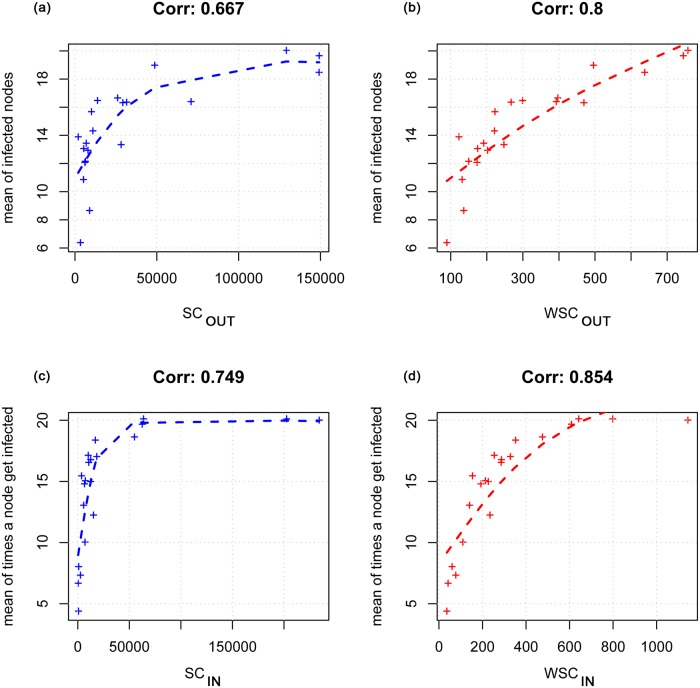
Correlation between model results and the strength centrality measures in the assumption that real values of population and number of moved animals are adopted. In the upper side of the figure the correlation between the node’s risk (the mean number of infected nodes) and SC_OUT_ (a) and WSC_OUT_ (b) is showed; the last two graphs report the correlation between node’s vulnerability (the mean number of times a node gets infected) and the SC_IN_ (c) and WSC_IN_(d).

Fig 7 shows the correlation values between model results and the strength centrality measures when real values of population and number of moved animals (second condition) are used. The correlation values are greater for the weighted measures in both risk of infection (Fig 7(b)) and vulnerability (Fig 7(d)) comparisons (0.8 and 0.85 respectively).

Table 2, following the example in Fig 4, reports data about incoming movements to Friuli Venezia Giulia region for years 2008–2010 (percentage values) Both in previous and successive years, the region tends to be related to the same regions and with the same intensity.

**Table 2:** Animals moved to Friuli-Venezia-Giulia region (percentages) in years 2008 to 2010.

	VENETO	LOMBARDY	SOUTH TYROL	TRENTINO	PIEDMONT	OTHER REGIONS
2008	81%	5%	7%	2%	2%	3%
**2009**	**82%**	**8%**	**7%**	**1%**	**1%**	**1%**
2010	81%	8%	6%	3%	1%	1%

Although Italian regions tend over time to trade with same regions and the same number of animal, at the least in percentages, the randomized network was considered and analyzed

**Fig 8:**
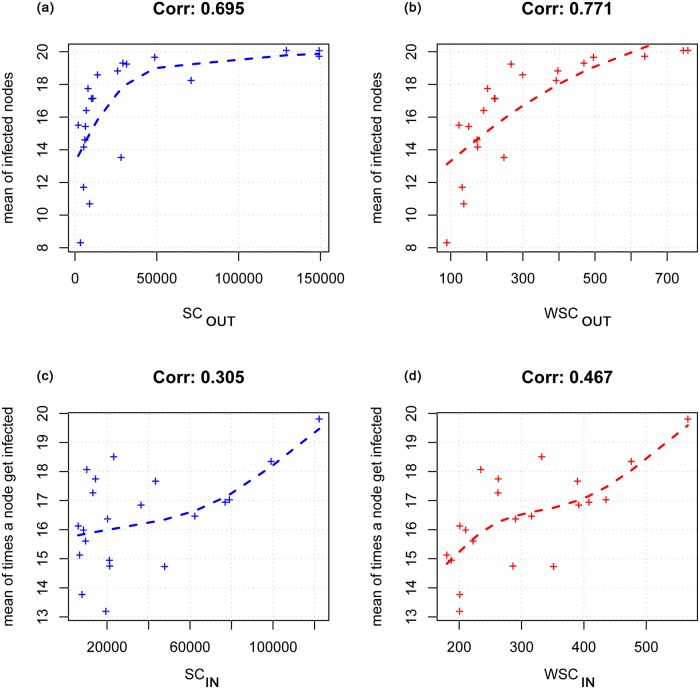
Correlation between model results and the strength centrality measures for the randomized network. In the upper side of the figure the correlation between the node’s risk (the mean number of infected nodes) and SC_OUT_ (a) and WSC_OUT_ (b) is showed; the last two graphs report the correlation between node’s vulnerability (the mean number of times a node gets infected) and the SC_IN_ (c) and WSC_IN_(d).

Fig 8 shows the correlation values between model results and the strength centrality measures for the randomized network (second condition). The correlation values are greater for the weighted measures in both risk of infection (Fig 8(b)) and vulnerability (Fig 8(d)) comparisons (0.7 and 0.45 respectively). It is worth to note that the correlation between the vulnerability and the measures of centrality (both classical and weighted) is lower than the association between the risk of infection and the weighted degree.

The identification of a new weighted degree measure arises from the need to consider the number of ties and tie weights simultaneously in order to correctly estimate the centrality of each actor within a weighted network. Opsahl et al 2010 proposed measures including a tuning parameter to control the relative importance of these two aspects. This parameter, varying from 0 to 1, needs to be subjectively set to give more importance either to the number of ties or to tie weights. To overcome the subjectivity of the use of this parameter, the new proposed weighted degree measure is objectively calculated. In the case study proposed in this work, the weighed degree is a number of ties that includes, on average, the 90% of the animals moved.

The worst case in terms of equal weight distribution occurs when all weights are equal to 1 for DC-1 links. The equation becomes: $AUC_{Fc}=\frac{1}{2}+\frac{1}{SC}\cdot \sum_{i=1}^{DC-1}i$

This highlights that varying SC and DC in their domain, different R values can be obtained, as reported in Table 3. When DC is equal to 2 the reduction factor R has a range between 0.5 and 0.8, never getting values less than 0.5; when DC approaches infinity, R approaches 0, this being the maximum difference between the standard and new weighted centrality measure.

**Table 3:** Limit values of R, varying SC and DC in their domain.

	DC = 2		DC = ∞	
	SC = ∞	SC = 3	SC>>DC	SC~DC
	min	max	min	max
AUC	0,5	0,8	0,5	<1
AUC_max_	1	1	∞	∞
**R**	**0,5**	**0,8**	**0**	**0**

Therefore the new weighted measure best captures the characteristics of a node in a highly connected weighted network.

As shown in Fig 5, the differences between the weighted and classical measures are even more pronounced for higher DC values. WDC could be used for the detection of high-risk holdings, both for contracting and spreading an infection within the network, and their removal, e.g. by trade restrictions or selective vaccination or culling, can efficiently change the network structure decomposing it into fragments so that infection chain is interrupted.

Although the region level network has been used throughout the paper, the Italian holding level network has been considered to evaluate node targeted selection based on proposed measures. Italian network at holding level consists of 122,702 nodes. In case of a disease control strategy based on targeted selection, centrality measures can be used. The selection of the nodes with degree value greater than the 95% percentile leads to 6,530 nodes using DC and 6,136 using WDC selection. The number of nodes common to both the measures are 5,148 nodes that correspond to a potential reduction of 21%.

A centrality measure that takes into account the heterogeneity of the connections within a real network of animal movements, allows then a more precise and accurate identification of the crucial nodes for the spread of an epidemic disease. This identification leads to a targeted surveillance with consequent reduction of costs and a better allocation of resources.
